# Combined Optical-Electrical Optimization of Cd_1−x_Zn_x_Te/Silicon Tandem Solar Cells

**DOI:** 10.3390/ma13081860

**Published:** 2020-04-15

**Authors:** Mehmet Koç, Giray Kartopu, Selcuk Yerci

**Affiliations:** 1 The Center for Solar Energy Research and Applications (GUNAM), Ankara 06800, Turkey; mehmetkoc.ee@gmail.com; 2 Micro and Nanotechnology Department, Middle East Technical University, Ankara 06800, Turkey; 3 Centre for Solar Energy Research, OpTIC, Swansea University, St. Asaph Business Park, Saint Asaph LL17 0JD, UK; giray.kartopu@swansea.ac.uk; 4 Department of Electrical and Electronics Engineering, Middle East Technical University, Ankara 06800, Turkey

**Keywords:** photovoltaic cell, tandem cell, CdTe, CdZnTe, ZnTe, c-Si, IBC silicon cell

## Abstract

Although the fundamental limits have been established for the single junction solar cells, tandem configurations are one of the promising approaches to surpass these limits. One of the candidates for the top cell absorber is CdTe, as the CdTe photovoltaic technology has significant advantages: stability, high performance, and relatively inexpensive. In addition, it is possible to tune the CdTe bandgap by introducing, for example, Zn into the composition, forming Cd_1−x_Zn_x_Te alloys, which can fulfill the Shockley–Queisser limit design criteria for tandem devices. The interdigitated back contact (IBC) silicon solar cells presented record high efficiencies recently, making them an attractive candidate for the rear cell. In this work, we present a combined optical and electrical optimization of Cd_1−x_Zn_x_Te/IBC Si tandem configurations. Optical and electrical loss mechanisms are addressed, and individual layers are optimized. Alternative electron transport layers and transparent conductive electrodes are discussed for maximizing the top cell and tandem efficiency.

## 1. Introduction

Record high efficiencies have recently been announced for many photovoltaic (PV) technologies (i.e., silicon, CdTe, CIGS, perovskite, organic, etc.) [1,2]. Although approaching their thermodynamic limits, also referred to as the Shockley–Queisser (SQ) limit, performance improvements of various types of solar cells have started to slow down. The SQ limit may be out of reach, as the SQ limit solely takes radiative recombination losses into account, whereas electrical and optical losses are still significantly reducing the performance. On the other hand, tandem-based solar cell configurations can surpass the SQ limits of single-junction solar cells. The SQ limit reaches a maximum for the two-junction tandem solar cells having a rear cell of silicon with the bandgap of 1.12 eV when a top cell with a bandgap of 1.81 eV is used [3]. Tandem cells with a two-terminal (2T) connection require continuous device fabrication and processing, a tunneling junction, and current matching of the subcells, all of which present challenges. For example, thermal stability of a subcell, which acts as the substrate for the tunneling junction and the other subcell, must be sufficiently high. The current matching requirement necessitates fine-tuning of the subcell parameters, which causes sacrifices in cell properties. On the other hand, a four-terminal (4T) tandem device would have fewer processing concerns as it involves attaching one cell on top of another with a transparent index-matching interlayer, thereby permitting the two cells to be produced and function totally independently in terms of their PV yields. Additionally, tolerance to the top cell bandgap increases compared to the 2T tandem configuration [4].

In recent years, both perovskite and III-V solar cells have been studied as a top cell partner to silicon. More than 25% conversion efficiencies are presented with various approaches such as conformal coating of the top cell on textured silicon rear cell and bandgap tuning of the top cell [5,6,7,8,9]. Regarding III-V/Si tandem cells, the most notable device was reported by Essig et al., where ~30% efficiency is achieved in 4T configuration using a ~18% GaInP top cell and ~12% Si rear cell in the tandem device [10]. However, both of these top cell candidates have their shortcomings in that the perovskite cells still suffer from instability (and cannot match the lifetime of silicon cells) and the III-V cells are produced only at small scale and yet at high cost. As an alternative, the mature CdTe thin film technology can present certain advantages, having an established long lifetime and relatively inexpensive PV solution with remarkable performance. Additionally, it is known that the introduction of Zn (or Mg) to CdTe can shift its bandgap from ~1.45 eV to higher energy levels, up to ~2.2 eV in the case of Zn alloying, following the relationship [11]

E_g_ (Cd_1−x_Zn_x_Te) = x E_g_ (CdTe) + (1 − x) E_g_ (ZnTe) − bx (1 − x) (1)

where E_g_ (CdTe) = 1.45 eV, E_g_ (ZnTe) = 2.2 eV, x is the Zn/(Zn + Cd) ratio, and b is the band bowing parameter.

In this work, we first carried out an optical optimization analysis of Cd_1−x_Zn_x_Te (denoted C_1−x_Z_x_T hereafter) top cells in tandem with an interdigitated back contact (IBC) c-Si rear cell based on various practical thickness and material combinations. To elucidate the effect of the top cell bandgap, various absorber compositions were investigated: CdTe, C_0.8_Z_0.2_T, C_0.6_Z_0.4_T, C_0.4_Z_0.6_T, C_0.2_Z_0.8_T, and ZnTe. Optical loss mechanisms are examined and addressed. Additionally, alternative functional (buffer and contact) materials are explored. In the second part, the optimum thicknesses deduced from optical analyses are employed to conduct electrical simulations. Significant conversion efficiency improvements have been presented by further tuning of the material types and parameters.

## 2. Materials and Methods

The transfer matrix method (TMM) is utilized in computations of reflection, transmission, and absorption of each individual layer of the top cell. Incident light is assumed to be scattering as Lambertian at the silicon interface. The ray tracing method is utilized to compute the paths of the scattered light until becoming fully extinct. The optical coefficients (n and k) of C_1−x_Z_x_T [12], Mg_x_Zn_1−x_O (MZO) [13], Cd_x_Zn_1−x_O (CZO) [14], cadmium sulfide (CdS) [15], ethylene/vinyl acetate (EVA) [16], silicon [17], glass [18], and indium-tin-oxide (ITO) [19] used in our calculations are taken from the literature. The refractive index spectra of molybdenum oxide (MoO_x_) and magnesium fluoride (MgF_2_) are obtained via spectroscopic ellipsometry measurements (SOPRA GES-5E) with a spectral range from 1.23 to 5 eV using thermally evaporated layers on a c-Si substrate at 70° incidence angle. SCAPS-1D is used for electrical simulations [20], and generation profiles are imported from optical simulations. Simulation parameters of solar cells used in SCAPS-1D are taken from the literature [21,22,23] and presented in Table S1. The four-terminal design (4T) is assumed for the tandem solar cell, whereby C_1−x_Z_x_T and IBC c-Si are used as top and rear solar cells, respectively.

## 3. Results

In this section, first, optical data will be provided to highlight the maximum achievable photocurrents with C_1−x_Z_x_T/Si tandem cells, in line with the SQ limit. Refined structures are then presented, addressing material related limitations to both optical and electrical tandem operations.

### 3.1. Optical Simulations

#### 3.1.1. The SQ Limit

**Figure 1:**
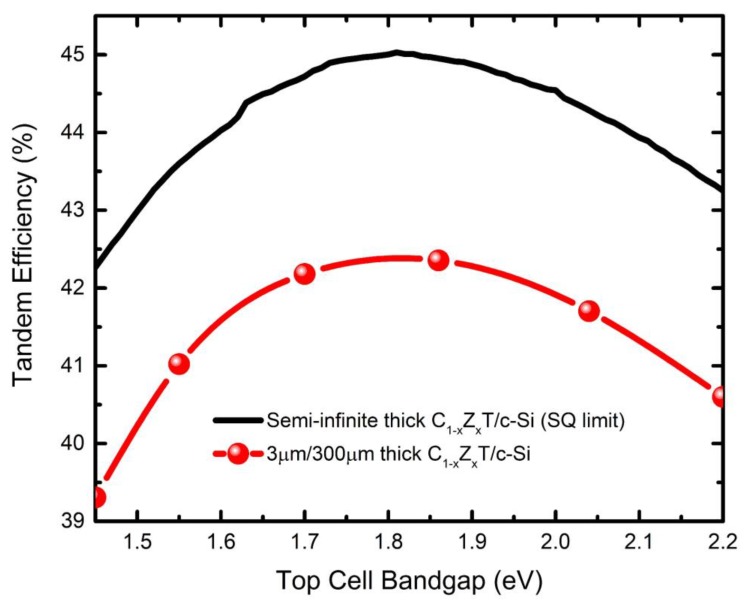
Maximum achievable conversion efficiency for C_1−x_Z_x_T/c-Si tandem cell calculated using the 4T tandem design. The two cases shown correspond to the SQ limit, which assumes semi-infinite absorber thicknesses and zero loss due to parasitic absorption, and a more practical limit, assuming finite thicknesses of 3 and 300 µm for the C_1−x_Z_x_T and c-Si absorbers, respectively, and the external reflection loss (i.e., due to air–C_1−x_Z_x_T interface) is eliminated.

Optically calculated C_1−x_Z_x_T/c-Si 4T tandem device performance as a function of the top cell bandgap is illustrated in Figure 1. In these calculations, reflection is taken as zero, and no functional layers (i.e., assuming only the two absorbers are stacked up) are employed. The maximum efficiency is obtained with C_0.4_Z_0.6_T as the top cell absorber, which has a bandgap of 1.86 eV. In a more practical example, the thicknesses of the C_1−x_Z_x_T and c-Si layers are reduced to 3 and 300 µm, respectively. The results are in good agreement with the SQ calculations with an offset difference in efficiency. The offset is ascribed to underutilization of incident light as a result of the practical extinction coefficients of absorbers, and the transmission.

#### 3.1.2. Full Device Structures

**Figure 2:**
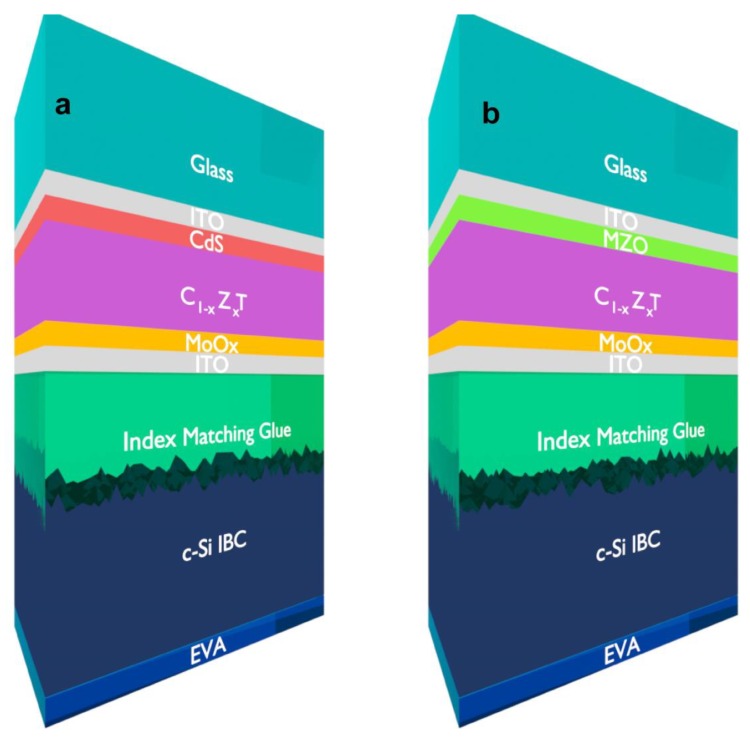
Schematic of C_1−x_Z_x_T/c-Si interdigitated back contact (IBC) tandem cells with top cell electron transport layers (ETLs) of CdS (**a**) and Mg_0.23_Zn_0.77_O (**b**).

In the more detailed optical simulations, CdS is used as an electron transport layer (ETL) for the top cell for most tandem configurations, being the traditional partner to the CdTe absorber. Additionally, magnesium zinc oxide (MZO) is employed as an ETL alternative. MoO_x_ is used as the hole transport layer (HTL) for the top cell. ITO is used as the transparent conducting oxide (TCO) on both sides of the top cell due to its good optical and electrical properties. The rear cell is assumed to be the IBC Si cell with 25.2% conversion efficiency. The refractive index of the index-matching glue, employed in between the two cells, is taken as 1.6 with zero extinction coefficient. Silicon nitride (SiN_x_) is utilized as the antireflection coating for the IBC Si cell. EVA is assumed to be used to protect the contacts of the IBC silicon cell. The optically simulated structures are illustrated in Figure 2. A performance parameter—the maximum achievable photocurrent (MAPC)—is defined to indicate the absorption in the cell and it can be calculated using Equation (2) [24]:

MAPC = q ∫ ϕ(λ)A(λ)dλ(2)

where q is the Coulomb charge, ϕ is photon flux in (photons cm^−2^ s^−1^ nm^−1^), and A is absorption in the active layer. The top cell layers (C_1−x_Z_x_T, ETL, HTL, and TCO layers) interact with the light in the specular domain, which is the result of the planar configuration. On the other hand, the IBC Si cell is assumed to scatter the incident light into the silicon in a Lambertian profile. Transmission, reflection, absorption, and external quantum efficiency (EQE) spectra of the rear cell are computed according to the study of Smith et al. [25].

**Figure 3:**
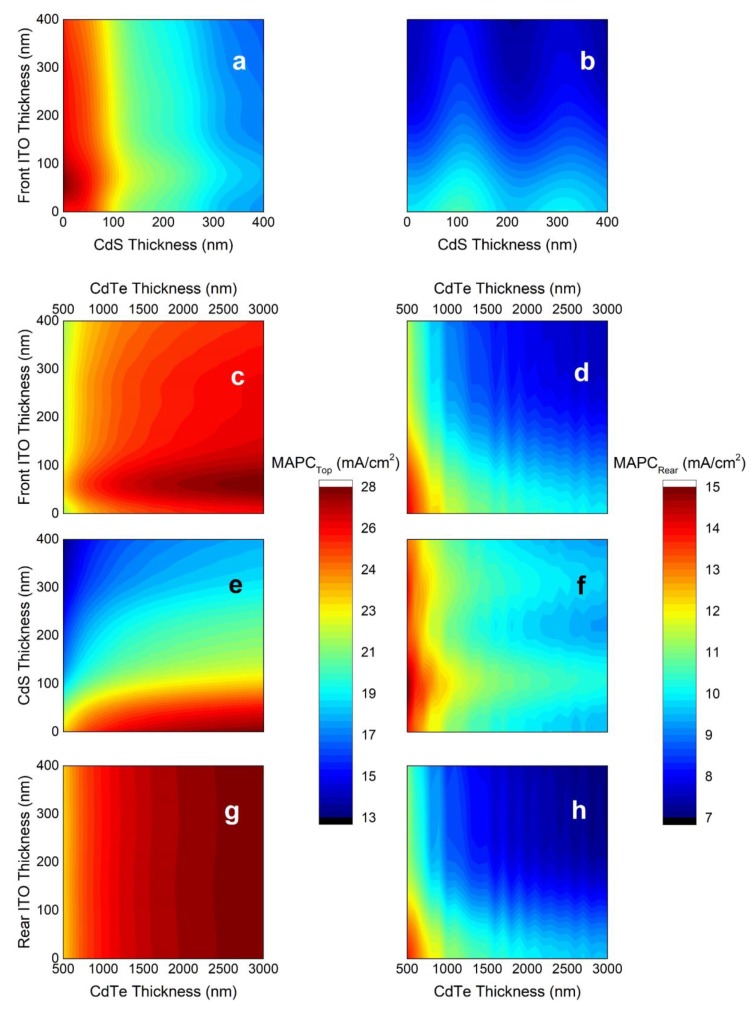
The maximum achievable photocurrent (MAPC) of CdTe and IBC Si cells for various front indium-tin-oxide (ITO) and CdS thicknesses for a constant CdTe thickness of 3000 nm, assuming no rear ITO layer is present (**a**,**b**); for various front ITO and CdTe thicknesses, assuming no CdS and rear ITO layers are present (**c**,**d**); for various CdS and CdTe thicknesses, assuming a front ITO layer thickness of 55 nm and no rear ITO is present (**e**,**f**); and for various rear ITO and CdTe thicknesses, assuming a front ITO layer thickness of 55 nm and no CdS layer is present (**g**,**h**), respectively.

As the practical thickness range of the CdTe layer for an efficient CdTe cell is 0.5 to 3.0 µm [26], most of the incident light is absorbed within a single pass (Figure S1). Thus, the interference of light due to the CdTe absorber is insignificant. The effect of thicknesses of the front layers, CdS and ITO, on the MAPCs of CdTe and IBC Si cells are presented in Figure 3a,b, respectively. To investigate the effects of just the front layers, a sufficiently thick (3 µm) CdTe and no rear ITO are assumed. MAPC of the CdTe cell presents a local maximum at approximately 55 nm thick front ITO layers (Figure 3a), whereas any increased thickness of the front ITO leads to parasitic loss and reduces the MAPC of the IBC Si cell (Figure 3b) [24]. This can be ascribed to the strong free carrier absorption and low refractive index of the ITO layer at the long wavelengths where incident light reaches the IBC Si cell. The CdS layer has adverse effects on the MAPC of the CdTe cell, regarding its nonzero extinction coefficient in the UV and visible parts of the spectrum (Figure S2), whereas it presents local maxima at odd integer multiples of 105 nm in the MAPC of IBC Si cell. These distinct constructive interference thicknesses correspond to the quarter-wave optical thicknesses (QWOT) of the CdS layer at a wavelength of 900 nm. This wavelength is related to the absorption onset of the CdTe absorber, where incident light flux reaches its peak for the rear cell.

The variations of the MAPCs of the CdTe and Si IBC cells with front ITO, CdS, CdTe, and rear ITO thicknesses are investigated in Figure 3c,e,g and Figure 3d,f,h, respectively. Although the optimum front ITO thickness for the MAPC of the CdTe cell is 55 nm irrespective of the CdTe thickness (Figure 3c), it leads to parasitic absorption in the IBC Si cell for any thickness of the CdTe layer as shown in Figure 3d. Similarly, the parasitic behavior of the CdS layer on the CdTe cell and the optimum thickness of the CdS (i.e., QWOT condition) to achieve a local maximum in the MAPC of the IBC Si cell are valid at different CdTe thicknesses, as can be seen in Figure 3e,f. Finally, although the rear ITO shows almost no effect on the MAPC of the CdTe cell, it leads to parasitic loss in MAPC of the IBC Si cell as shown in Figure 3g,h. The parasitic loss due to the rear ITO, similar to the front ITO, is due to its nonzero extinction coefficient in the near infrared (IR) as a result of high free carrier concentration (Figure S2).

**Figure 4:**
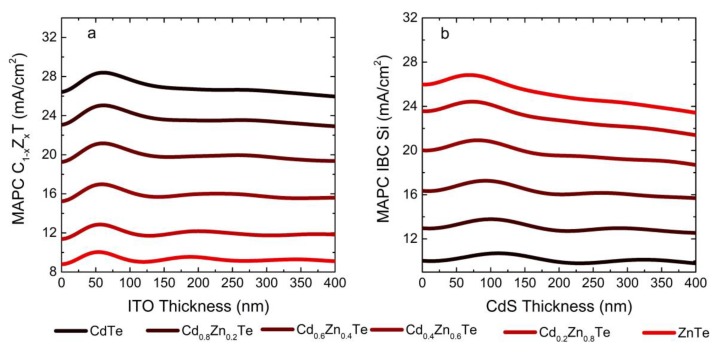
The MAPC of C_1−x_Z_x_T (**a**) and IBC Si (**b**) cells for various x values and C_1−x_Z_x_T thickness of 3 μm. In panel (**a**), the front ITO thickness is varied with no CdS and no rear ITO layers employed, whereas in panel (**b**), the CdS thickness is varied with 55 nm front ITO and no rear ITO layer employed.

The optimum front ITO thickness of 55 nm found for the CdTe top cell is also valid for C_1−x_Z_x_T absorbers regardless of the Zn content (Figure 4a). Note that both front and rear ITO layers absorb in the near IR and therefore reduce the MAPC of the Si IBC cell irrespective of the C_1−x_Z_x_T composition. Although absorption loss in CdS reduces the MAPC of the C_1−x_Z_x_T cell, the QWOT condition yielding maximum at the MAPC of the IBC Si cell is also valid for any Zn content. Obviously, a thinner CdS layer satisfies the QWOT condition with increasing Zn content as the absorption onset shifts to shorter wavelengths (Figure 4b).

**Figure 5:**
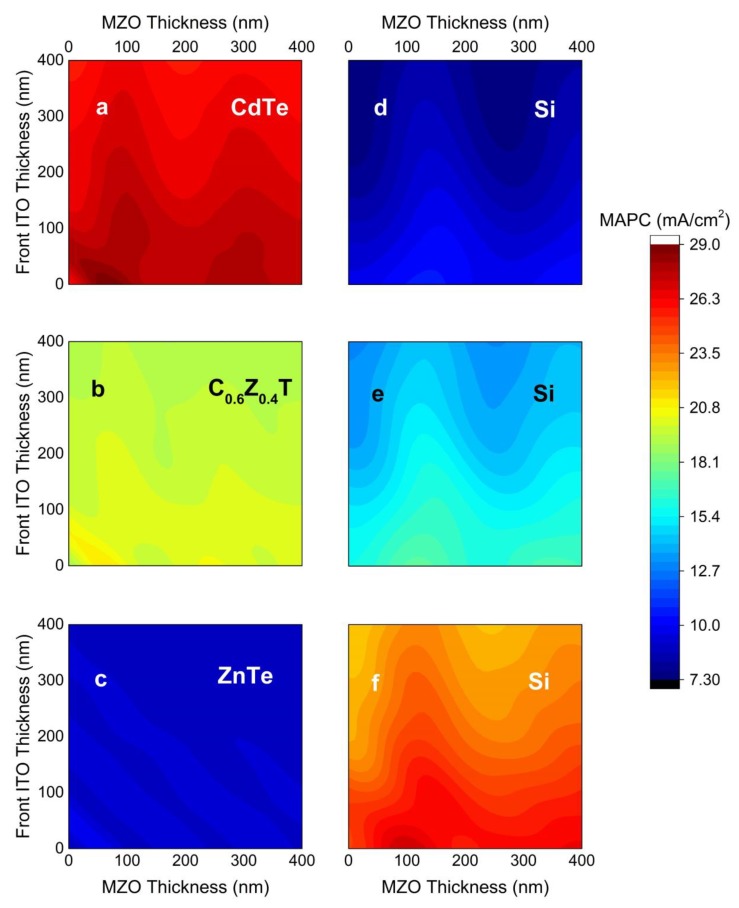
MAPC of CdTe (**a**), C_0.6_Z_0.4_T (**b**), and ZnTe (**c**) top cells, along with the corresponding IBC Si cells (**d**–**f**) as a function of front ITO and MZO thicknesses. In all cases, C_1−x_Z_x_T = 3 µm, HTL (MoO_x_) = 5 nm, and no rear ITO is used to isolate the effect of the front ITO and MZO layers.

Mg_x_Zn_1−x_O (0 < x < 0.4) is commonly considered as a replacement for CdS to boost the photocurrent of CdTe thin film solar cells [27,28,29] due to its much smaller extinction coefficient (Figure S2). To investigate the effect of an MZO (Mg_0.23_Zn_0.77_O) ETL, the MAPCs of CdTe, C_0.6_Z_0.4_T, and ZnTe top cells and IBC Si rear cell with respect to ITO and MZO thicknesses are shown in Figure 5. The refractive index of MZO is adjacent to that of ITO in the visible, and as a result, MAPC of the top cell presents local maxima for total thicknesses of front ITO (t_ITO_) and MZO (t_MZO_) layers (i.e., t_ITO_ + t_MZO_ ≈ 60 nm). Another local maximum trend is observed for odd integer multiples of MZO thickness of ~100 nm for CdTe and C_0.6_Z_0.4_T absorbers (Figure 5a,b). This thickness corresponds to QWOT at the 700–800 nm wavelength region of the spectrum. As the refractive index of ITO is adjacent to glass in this region of the spectrum, the consolidated thickness behavior is no longer valid, thus the interference of incident light is solely defined by MZO thickness. These anti-reflection trends observed for odd multiples of MZO thicknesses shift to thinner values with increasing Zn content as the absorption edge shifts to shorter wavelengths (Figure 5b). As the absorption edge moves to shorter wavelengths with Zn content, the local maxima when the condition of odd multiples of t_ITO_ + t_MZO_ dominates. Therefore, the local maxima of the MAPC for ZnTe present inclined lines, as shown in Figure 5c. Similar to the case of CdS ETL (Figure 4b), the MAPC of the IBC Si cell displays local maxima due to constructive interferences in the MZO layers. MZO presents QWOT behavior for ~120 nm for the MAPC of the IBC Si cell (Figure 5d), which shifts to thinner values with increasing Zn content as shown in Figure 5e,f.

The parasitic absorption in the CdS layer can also be eliminated by flipping the C_1−x_Z_x_T solar cell upside down. In related simulations, we assumed the top cell is covered with a humidity blocking encapsulant (e.g., EVA), as shown in Figure S3. This inverted configuration can eliminate the parasitic absorption of the CdS layer as most of the incident photons on C_1−x_Z_x_T layer are absorbed within a single pass up to the 600 nm wavelength (Figure S1), and CdS has almost zero extinction coefficient for wavelengths longer than 550 nm. Additionally, QWOT of the CdS layer can be utilized without any parasitic effect on the top cell.

**Figure 6:**
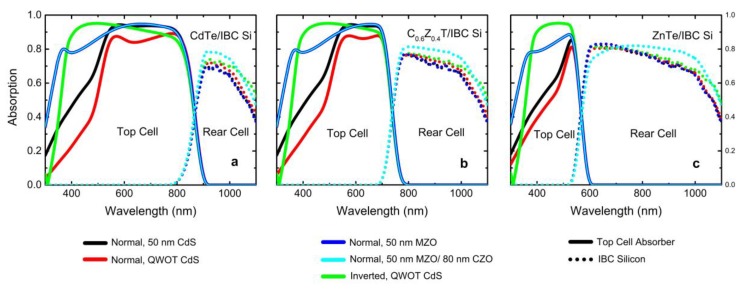
Absorption spectra of CdTe (**a**), C_0.6_Z_0.4_T (**b**), and ZnTe (**c**) top cells (solid lines), along with the corresponding IBC Si rear cell absorption spectra (dotted lines); in the normal configuration with 50 nm CdS (black), QWOT CdS (red), 50 nm MZO (blue), 50 nm MZO, and 80 nm CZO as rear TCO (cyan) and in the inverted configuration with QWOT CdS (green). In all cases, C_1−x_Z_x_T = 3 µm, MoO_x_ = 5 nm, front ITO = 55 nm, and rear TCO (ITO or CZO) = 100 nm. QWOT CdS values are 105 nm for CdTe, 95 nm for C_0.6_Z_0.4_T, and 65 nm for ZnTe top cells.

The absorption spectra of the optimized normal top cell configuration with a CdS or Mg_0.23_Zn_0.77_O window ETL and the inverted design with a CdS rear ETL are given in Figure 6a–c, and the calculated MAPCs for top and rear cells are given in Table 1. Note that a 110 nm thick MgF_2_ at the front is used as an anti-reflective coating (ARC). Significant improvements in MAPC of the top cell can be achieved when the MZO is used instead of CdS in the normal configuration. For example, the MAPC of the top cell with CdTe absorber is 1.78 mA/cm^2^ higher with MZO compared with CdS having the same thickness (50 nm). Improvement in the CdTe cell is 1.69 mA/cm^2^ for the inverted structure with QWOT ETL. In the case of the C_0.6_Z_0.4_T absorber, the MAPC improvement is 2.08 mA/cm^2^ for the inverted design and 1.79 mA/cm^2^ for the MZO replacement in the normal configuration relative to the CdS as ETL. Finally, in the case of the ZnTe absorber, the enhancement in the top cell MAPC is 2.36 mA/cm^2^ for the inverted design and 1.74 mA/cm^2^ for the MZO replacement relative to the CdS as window ETL. The change in the MAPC of the IBC Si cell is a decrease of approximately 0.6 mA/cm^2^ with the MZO replacement for all cases. Utilization of the CdS layer with QWOT value results in a less than 0.35 mA/cm^2^ increase in the MAPC of the rear cell; however, it causes more than a 3 mA/cm^2^ MAPC penalty at the top cell. In the meantime, more than 1 mA/cm^2^ MAPC improvements are observed for the rear cell in the inverted structure cases.

The rear cell suffers optically; particularly, transmitted light to the rear cell is reduced. The transmitted light is decreasing with increasing wavelength as a result of the unfavorable (low) refractive index alignment for long wavelengths and free carrier absorption in the front and rear ITO layers, which are due to the high doping concentration in the ITO. As the rear ITO acts parasitic for the rear cell and has no effect on the top cell, replacing it with an alternative material having a higher refractive index for long wavelengths (λ > 800 nm) and lower free carrier concentration can eliminate this parasitic loss in a very effective way with no optical trade-off. Cd_0.9_Zn_0.1_O (CZO), for example, fulfills both criteria, where it has a higher refractive index of ~2.12 for λ > 800 nm and a slightly smaller extinction coefficient for the same region compared to ITO. The absorption spectra of the selected top cell absorbers with an MZO transport layer replacement and a CZO rear TCO replacement are also presented in Figure 6. It can be seen that absorption spectra of the rear cell in both cases improve significantly. This is ascribed to optically better aligned refractive index profile and reduced parasitic absorption within the CZO layer. Up to 1.77 mA/cm^2^ greater MAPC can be achieved in the rear cell with the combined use of MZO and CZO in place of CdS and ITO, respectively.

### 3.2. Electrical Simulations

Device simulation results for the normal configuration with 55 nm front ITO, 50 nm CdS ETL, and 3 μm C_1−x_Z_x_T, as a function of the Zn ratio, are summarized in Table 2. The spatial photoelectron generation profile is simulated optically, which is then imported to the electrical simulations. To present practical devices with a polycrystalline absorber, defect mechanisms are introduced in electrical simulations, such as surface and bulk recombination in the top cell absorber and transport layers (a lifetime of 10 ns is assumed for MZO and CdS), in agreement with experimental results [21,22,23]. The minority carrier lifetime is assumed to be 5 ns in top cell absorbers, and surface recombination velocity at the ETL/top cell absorber interface is taken as 500 cm/s. Series and shunt resistances are taken as 2 Ω/cm^2^ and 2 kΩ/cm^2^, respectively. The highest tandem conversion efficiency among various C_1−x_Z_x_T compositions is computed with the case of x = 0.2, as 22.9%. The top cell conversion efficiency is 16.9%, which is significantly below the recent record for CdTe solar cells: 22.1%. The inferior top cell performance is caused mainly by (i) the significant parasitic absorption in the CdS layer, causing a MAPC loss of around 2 mA/cm^2^, and (ii) the reduced V_OC_ driven by increased recombination as a result of the unfavorable energy offset at the CdS/C_1−x_Z_x_T interface [27,30]. For the record efficiency cells, more transparent ETLs, such as Mg_x_Zn_1−x_O, and Se alloying of the CdTe absorber (to reduce the bandgap towards 1.35 eV) are utilized, which enhances the current collection at short and long wavelengths, respectively. Further, the band alignment between the ETL/absorber can be fine-tuned by the Mg and Se content in the respective layers, which suppresses interfacial recombination, thereby increasing the Voc. Similarly, the corresponding IBC Si cell conversion efficiency, 6.61%, is suboptimal. The main reason for the performance undercut is the weak transmittance of long wavelength photons to the IBC Si cell.

The CdTe/IBC Si tandem cell efficiencies as a function of minority carrier lifetime are given in Figure S4a for three different surface recombination velocities: 10, 50, and 500 cm/s. Minority carrier lifetime increased from 1 to 100 ns yields an efficiency improvement of 0.55%, whereas decreasing the surface recombination velocity from 500 to 10 cm/s produces 0.20% more tandem efficiency. However, it is not enough for the CdTe/IBC Si tandem cell to surpass the IBC Si single junction cell efficiency. Additionally, the effect of series and shunt resistances on the tandem cell efficiency is presented in Figure S4b. To generate further improvements, the electrical device parameters can be refined. For instance, increasing the shunt resistance to 4 kΩ/cm^2^ and the minority carrier lifetime to 100 ns results in more than 1% gain in tandem cell efficiency.

Device simulation results for the MZO ETL replacement model are summarized in Table 3. All thicknesses are kept unchanged. Similar to the CdS ETL model, the spatial photoelectron generation profile is calculated optically. The most pronounced result is that the highest tandem conversion efficiency, 24.9%, is 2.9% higher than the CdS ETL case with the same thickness configuration. This performance improvement is only computed for the top cell conversion efficiency as expected, as the CdS layer is detrimental exclusively for the top cell. The improvement in V_OC_ is 86 mV for the champion cell, which uses a C_0.6_Z_0.4_T top cell absorber.

**Figure 7:**
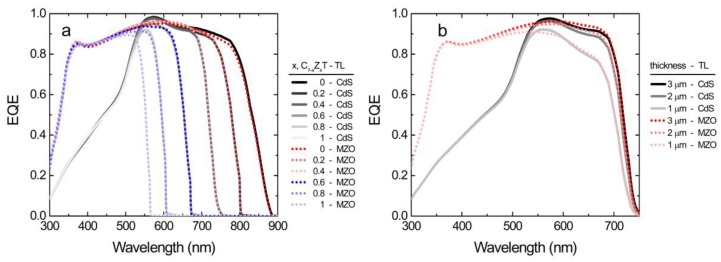
Calculated external quantum efficiency (EQE) spectra for the C_1−x_Z_x_T top cell as function of (**a**) the absorber composition (x = 0, 0.2, 0.4, 0.6, 0.8, and 1) at 3 µm thickness and (**b**) the absorber thickness (1, 2, and 3 μm) at x = 0.6 for with 50 nm ETL of CdS and MZO.

External quantum efficiency (EQE) response of the C_1−x_Z_x_T top cell for various absorber compositions and 50 nm CdS and MZO ETLs is provided in Figure 7a. The long wavelength cut-off corresponds to the absorber bandgap, and therefore shows a progressive blue shift from ~850 nm to ~550 nm with increasing Zn concentration, also narrowing the response width. The short wavelength response is dominated by the ETL absorption and MZO can be seen to deliver much superior the UV-blue response compared to CdS. The effect of the absorber thickness on the EQE spectrum is illustrated in Figure 7b for the absorber composition C_0.6_Z_0.4_T. The red response (>500 nm region) becomes weaker especially at 1 μm thickness, causing significant photocurrent losses. The overall EQE response saturates at absorber thickness of ~3 μm.

**Figure 8:**
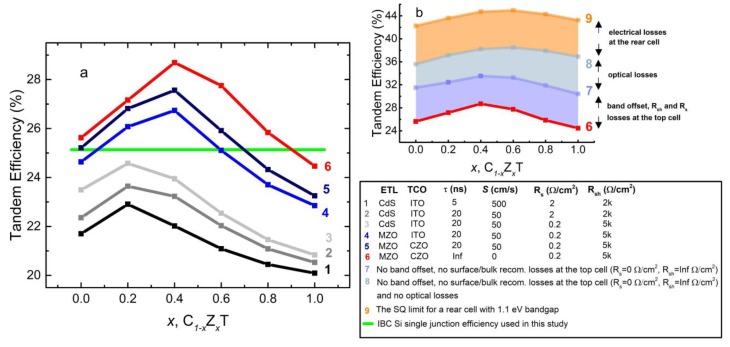
(**a**) C_1−x_Z_x_T/IBC Si tandem efficiency as a function of various *x* values for different ETL (CdS and MZO) and rear TCO (ITO and CZO) combinations, along with different shunt-series resistances (2000–2 and 5000–0.2 Ω/cm^2^), minority carrier lifetimes (5 and 20 ns), and surface recombination velocities (50 and 500 cm/s): Curves 1–5. Curve 6 is the case for the eliminated bulk and surface recombination at the top cell with MZO ETL and CZO rear TCO for R_s_ = 0.2 and R_sh_ = 5 kΩ/cm^2^. The single junction efficiency of IBC Si cell, 25.2 %, is illustrated as a reference (horizontal green line). (**b**) The estimated share of efficiency losses with respect to the SQ limit. Curve 7 represents the most ideal electrical case for the top cell with no series/shunt resistance losses and no energy band offset at interfaces, tailored for each absorber composition. Curve 8 represents the ideal electrical case for the top cell and eliminated optical losses, parasitic absorptions, reflection, and transmission for the both cells. Curve 9 is the SQ limit for the double-junction tandem solar cell with a rear cell with 1.1 eV bandgap.

The C_1−x_Z_x_T/IBC Si tandem cell efficiencies with various defect mechanisms and device parameters combinations are presented in Figure 8a along with different ETL/rear TCO configurations. The single junction efficiency of the IBC Si solar cell, 25.2% (horizontal line), is also given for benchmarking. The Zn content which gives maximum tandem cell efficiency shifts from 0.2 to 0.4 with MZO-CdS replacement (curves 1–6). Trends for maximum tandem cell efficiency stay unchanged with improving defect mechanisms. It is demonstrated that cases which surpass the single junction efficiency of the IBC Si cell have MZO as the ETL, a minority carrier lifetime of 20 ns and a surface recombination velocity of 50 cm/s. When the rear TCO is selected as CZO, CdTe, C_0.8_Z_0.2_T, C_0.6_Z_0.4_T, and C_0.4_Z_0.6_T top cells are the ones that can outweigh the IBC Si single cell, whereas in the case of ITO, they are C_0.8_Z_0.2_T and C_0.6_Z_0.4_T.

Electrically calculated, more idealized cases (curves 7 and 8) and the SQ limit (with no optical or electrical losses, curve 9) are compared in Figure 8b. Trends in tandem cell efficiency with Zn content stay unchanged with electrical idealization of the top cell (curve 7), whereas an offset performance improvement is demonstrated for both CZO and ITO as rear TCO cases. In the case of the optical idealization (curve 8), the Zn content that gives the maximum tandem cell efficiency shifts to 0.6. When assuming perfect energy alignment of CB and VB levels, perfect front and rear contact energy alignment and no bulk and surface defects and no series and shunt resistance limitations (curve 7), the only loss due to the top cell is the optical ones, i.e., parasitic absorption and reflection losses. The best tandem performance is ~11% short of the SQ limit. Provided the optical losses due to the top cell are also eliminated (curve 8), the deficiency with respect to the SQ limit reduces to ~6%, which should account for losses due to the IBC Si rear cell.

Device simulation results with an 80 nm thick CZO, employed as rear TCO in place of ITO, are presented in Table 4, where all other thicknesses and layers are kept as unchanged. It can be seen from the results that almost 1% further conversion efficiency improvement is obtained with this replacement and improvement is exclusive for the IBC Si cell, as predicted.

The J–V curves of the selected C_1−x_Z_x_T cases, x = 0.4 and 0.6, from Table 2 and Table 3 are presented in Figure S5.

## 4. Discussion

The gap between the SQ limit and the electrical device simulations (Figure 8b) is seen to be dominated by electrical and optical losses in the top cell as well as losses due to the rear IBC Si cell, which was not optimized for the IR response. Improvements towards reducing parasitic absorption (at the transport and TCO layers) and reflection losses are still required to reduce this gap. Utilization of a bifacial silicon solar cell, on the other hand, can provide an additional boost, especially when high bifaciality and albedo value are achieved. Moreover, further improvements in the tandem cell efficiency can be achieved if light trapping schemes (additional to use of the MgF_2_ AR layer) can be introduced to the top cell, e.g., by conformal fabrication of the top cell over the textured IBC Si rear cell. This would present advantages at the top cell such as eliminated reflection losses, in particular at the near IR, and enhanced optical paths of the incident photons. Gaining on the IR losses would boost light capture by the IBC Si rear cell while the optical path enhancement would enable the use of thinner active layers without an optical trade-off in the top cell, which yields improved optical and electrical properties.

It is evident from both the optically and electrically simulated data that surpassing the IBC Si single junction efficiency is possible using a C_1−x_Z_x_T top cell in tandem. c-Si PV is a mature technology that allows adoptability to the tandem application. The 4T configuration also has the added advantages of independent fabrication and operation of subcells without the need of a tunneling (recombination) layer and current matching. However, the state-of-the-art with C_1−x_Z_x_T solar cells must be reviewed to address practicality and potential issues with the fabrication of high performance C_1−x_Z_x_T top cell. In terms of the bandgap, C_1−x_Z_x_T compositions with x = 0.2–0.6 are the most promising (see Figure 8), and CdTe is less favorable. For polycrystalline C_1−x_Z_x_T devices, there are no outstanding examples published hitherto, the major issue being the substantial Zn loss through the absorber passivation step that involves CdCl_2_ heat treatment (CHT). The highest performance reported using CHT-passivated C_1−x_Z_x_T has been limited to below 7% [31,32]. It is believed that Zn forms a volatile ZnCl_2_ compound and leaves the device, as no Zn can be found within the structure following the CHT. This step not only reverts the absorber composition to CdTe, but also makes it defective. Most recently, the record efficiency using a polycrystalline C_1−x_Z_x_T absorber was increased to 8.5% with a glass/ITO/CdS/CZT/Au cell structure, whereby the absorber was annealed in an argon–oxygen mixture at 400 °C without any chlorine being present, despite this annealing also causing some Zn loss and reduction of the bandgap (from ~1.83 to 1.71 eV) [33]. On the other hand, for epitaxial films and single crystals of C_1−x_Z_x_T, higher efficiencies can be achieved without the need of grain passivation. For example, Carmody et al. achieved epitaxial growth of 1.8 eV bandgap C_1−x_Z_x_T material on a Si (211) substrate and obtained 16% efficiency in single cell operation and 17% in 2T tandem with the Si substrate as the rear cell [34]. Therefore, epitaxial C_1−x_Z_x_T cells appear to be the most promising option for the short-term realization of high-performance 4T C_1−x_Z_x_T/Si tandem cells. However, the high cost associated with epitaxy substrate (e.g., GaAs, CdTe, and InSb) must be alleviated by the epitaxial lift-off (ELO) of the II–VI cell and reuse of the substrate, in an analogous manner to the more mature III–V solar cells [35,36].

## 5. Conclusions

We carried out optical and electrical simulations to evaluate the potential and limitations of C_1−x_Z_x_T/c-Si tandem solar cells in 4T configuration. Scenarios in which this efficiency limit can be exceeded were pursued using variable absorber band gaps and transport layers in the top cell.

We found the following:

1. The large extinction coefficient of CdS in the UV and visible impedes the usage of the CdS layer as a transparent electron transport layer in the normal top cell structure.
2. MZO replacement of CdS boosts the performance of the top and tandem cells significantly. Smaller and less dispersed extinction coefficient and the favorable energy level alignment provide J_SC_ and V_OC_ enhancements. Yet, there is still room for improvement. MZO has a refractive index (n) of ~1.9 in the UV–visible regions; replacing it with a higher refractive index material can boost the MAPC of the top cell significantly.
3. As an alternative to substitution of the CdS ETL, it is presented that optical performance of the tandem stack can be boosted by flipping the fabrication order while using an undoped absorber with the top cell. The main benefits of the proposed inverted configuration are that (i) the impact of parasitic absorption in CdS ETL layer on the top cell stack is wholly eliminated and (ii) improvements in MAPC of IBC Si cell caused by the constructive interference in CdS layer become more prominent.
4. To improve MAPC of IBC Si cell, transparent conducting electrodes (TCEs) with higher IR transparency should be utilized. Cd_0.9_Zn_0.1_O is suggested as a more efficient TCE in place of the rear ITO to fulfill these criteria. The higher refractive index of Cd_0.9_Zn_0.1_O yields a smoother transition towards the rear cell, and as a result, up to 4.33% tandem efficiency improvement becomes possible.

There is no interdependency between the front and rear ITO or the front ITO and CdS ETL, in terms of MAPC of the C_1−x_Z_x_T cell. MAPC of IBC Si cell shows a decreasing trend with the thickness of the front ITO, in respect of the increased reflection and free carrier absorption, and meanwhile it shows local maxima around the distinct CdS thickness, defined by the QWOT at the absorption onset wavelength of the top cell absorber. In the proposed inverted configuration, parasitic absorption of the CdS layer is eliminated. Additionally, as CdS has zero extinction coefficient for the interested part of the spectrum for the IBC Si cell, it leads to an anti-reflective behavior without any parasitic effect. Rear ITO behaves as a parasitic absorber for the IBC Si cell and it has no effect on MAPC of the C_1−x_Z_x_T cell. Employing a TCO with a higher refractive index and lower extinction coefficient at the long wavelengths improves MAPC of the rear cell and so the tandem performance significantly. Overall, this article paves a new avenue for the high efficiency CZT/c-Si tandem solar cells.

## Figures and Tables

**Table 1:** The MAPC (mA/cm^2^) of the top and rear cells for the optimized normal design, with CdS and Mg_0.23_Zn_0.77_O ETL window layers, and the ITO and CZO rear TCO layers and the proposed inverted design. CdS QWOT thicknesses are 105, 90, and 65 nm for CdTe, C_0.6_Z_0.4_T, and ZnTe, respectively; 110 nm MgF_2_ is used as anti-reflective coating (ARC). Thickness of top cell absorber and rear TCO are set to 3 µm and 80 nm, respectively.

Cell Structure TL/Thickness Rear TCO	Normal CdS/50 nm ITO	Normal CdS/QWOT ITO	Normal MZO/50 nm ITO	Normal MZO/50 nm CZO	Inverted CdS/QWOT ITO
Top Cell 1-CdTe	26.4	23.3	28.2	28.1	28.1
Rear Cell-Si	9.7	10.1	9.1	10.3	10.2
Top Cell 2-C0.6Z0.4T	18.8	16.5	20.6	20.5	20.8
Rear Cell 2-Si	16.4	16.9	15.8	17.4	16.9
Top Cell 3-ZnTe	7.3	6.6	9.1	9.1	9.7
Rear Cell 3-Si	27.1	27.3	26.5	27.4	27.3

**Table 2:** Electrical and optical device simulation data for a normal top cell structure with 50 nm CdS ETL, and the corresponding IBC Si rear and tandem cell results as a function of the Zn content in C_1−x_Z_x_T. Series and shunt resistances are used as 2 and 2 kΩ/cm^2^. Minority carrier lifetime is assumed to be 5 ns in top cell absorbers and surface recombination velocity at the ETL/top cell absorber interface is taken as 500 cm/s.

C_1−*x*_Z_*x*_T (*x*)	E_g_ (meV)	MAPC (mA/cm^2^)	J_SC_ (mA/cm^2^)	V_OC_ (mV)	FF (%)	η (%)	J_SC-Si_ (mA/cm^2^)	V_OC-Si_ (mV)	FF_Si_ (%)	η_Si_ (%)	η_Tandem_ (%)
0	1450	26.4	23.8	902	77.8	16.9	8.35	696	82.1	4.8	21.7
0.2	1550	22.8	20.6	996	79.2	16.3	11.43	704	82.2	6.6	22.9
0.4	1700	18.8	17.2	1038	74.1	13.2	15.02	711	82.3	8.8	22.0
0.6	1860	14.4	13.0	1025	74.0	9.8	19.04	717	82.4	11.3	21.1
0.8	2040	10.2	9.2	1008	73.5	6.8	22.93	722	82.5	13.7	20.4
1	2200	7.3	6.6	991	72.5	4.8	25.65	725	82.5	15.3	20.1

**Table 3:** Electrical and optical device simulation data for a normal top cell structure with 50 nm Mg_0.23_Zn_0.77_O as ETL, and the corresponding IBC Si rear and tandem cell structures as a function of the Zn concentration in C_1−x_Z_x_T. Series and shunt resistances are used as 2 and 2000 Ω/cm^2^. The minority carrier lifetime is assumed to be 5 ns in top cell absorbers and surface recombination velocity at the ETL/top cell absorber interface is taken as 500 cm/s.

C_1−*x*_Z_*x*_T (*x*)	E_g_ (meV)	MAPC (mA/cm^2^)	J_SC_ (mA/cm^2^)	V_OC_ (mV)	FF (%)	η (%)	J_SC-Si_ (mA/cm^2^)	V_OC-Si_ (mV)	FF_Si_ (%)	η_Si_ (%)	η_Tandem_ (%)
0	1450	28.2	25.1	904	78.7	17.9	7.9	694	82.1	4.5	22.4
0.2	1550	24.7	22.4	1000	80.7	18.1	11.0	703	82.2	6.4	24.5
0.4	1700	20.6	19.0	1124	76.1	16.3	14.6	710	82.3	8.6	24.9
0.6	1860	16.2	14.7	1136	74.7	12.5	18.7	716	82.4	11.0	23.5
0.8	2040	11.9	10.9	1122	74.4	9.1	22.6	721	82.5	13.5	22.6
1	2200	9.1	8.4	1109	73.8	6.8	25.3	724	82.5	15.1	21.9

**Table 4:** Electrical and optical device simulation data for a normal top cell structure with 50 nm Mg_0.23_Zn_0.77_O as ETL and 80 nm Cd_0.9_Zn_0.1_O as rear TCO, and the corresponding IBC rear and tandem cell structures as a function of the Zn content in C_1−x_Z_x_T. Series and shunt resistances are used as 2 and 2000 Ω/cm^2^. Minority carrier lifetime is assumed to be 5 ns in top cell absorbers and surface recombination velocity at the ETL/top cell absorber interface is taken as 500 cm/s.

C_1−*x*_Z_*x*_T (*x*)	E_g_ (meV)	E_g_ (eV)	MAPC (mA/cm^2^)	J_SC_ (mA/cm^2^)	V_OC_ (mV)	FF (%)	η (%)	J_SC-Si_ (mA/cm^2^)	V_OC-Si_ (mV)	FF_Si_ (%)	η_Si_ (%)	η_Tandem_ (%)
0	1450	1.45	28.1	25.1	904	78.7	17.9	8.9	697	82.1	5.1	23.0
0.2	1550	1.55	24.6	22.4	1000	80.7	18.1	12.3	706	82.2	7.1	25.2
0.4	1700	1.7	20.5	19.0	1124	76.1	16.2	16.0	712	82.3	9.4	25.6
0.6	1860	1.86	16.1	14.7	1136	74.7	12.5	20.0	718	82.4	11.8	24.3
0.8	2040	2.04	11.9	10.9	1121	74.4	9.1	23.6	723	82.5	14.1	23.2
1	2200	2.2	9.1	8.4	1109	73.8	6.8	25.9	725	82.5	15.5	22.3

## Supplementary Materials

The following are available online at https://www.mdpi.com/1996-1944/13/8/1860/s1, Figure S1: Single-pass absorption spectra for 3 µm thick top cell active absorber (CdTe, C_0.6_Z_0.4_T and ZnTe) obeying the Beer-Lambert Law for the incident light as a function of wavelength, Figure S2: Refractive index (n) and extinction coefficient (k) of materials used in this study, Figure S3: Schematic of the tandem device with inverted top cell structure. Front and rear TCOs are ITO; ETL is CdS; HTL is MoO_x_; and an index matching glue is employed between two cells. EVA is used to protect IBC Si cell contacts; glass is employed as the substrate, Figure S4: CdTe/IBC Si tandem efficiency as a function of (**a**) lifetime for three surface recombination velocities (10, 50 and 500 cm/s) for a 3 µm CdTe absorber and (**b**) series resistance for four shunt resistances (Infinite, 5k, 2k and 1k Ω/cm^2^) for a lifetime of 5 ns and surface recombination velocity of 500 cm/s. CdS is used as the ETL, Figure S5: Top cell J-V curves of selected C_1−x_Z_x_T cases (x = 0.4, 0.6) with 50 nm ETL of CdS and MZO, Table S1: Simulation parameters of solar cells used in SCAPS 1-D.
