# Supplementary material for: Combined Optical-Electrical Optimization of Cd1−xZnxTe/Silicon Tandem Solar Cells
Source: Materials (Basel). 2020 Apr 15;13(8):1860. doi: 10.3390/ma13081860 (PMC7215639; doi:10.3390/ma13081860)
Supplement: Supplementary file 1 [file materials-13-01860-s001.pdf]

Article

Combined Optical-Electrical Optimization of $Cd_{1-x}Zn_xTe$/Silicon Tandem Solar Cells

Mehmet Koç [1,2], Giray Kartopu [3] and Selcuk Yerci [1,2,4,*]

[1] The Center for Solar Energy Research and Applications (GUNAM), Ankara 06800, Turkey; mehmetkoc.ee@gmail.com
[2] Micro and Nanotechnology Department, Middle East Technical University, Ankara 06800, Turkey
[3] Centre for Solar Energy Research, OpTIC, Swansea University, St. Asaph Business Park, LL17 0JD, UK; giray.kartopu@swansea.ac.uk
[4] Department of Electrical and Electronics Engineering, Middle East Technical University, Ankara 06800, Turkey
* Correspondence: syerci@metu.edu.tr; Tel.: +90 (312) 210-2344

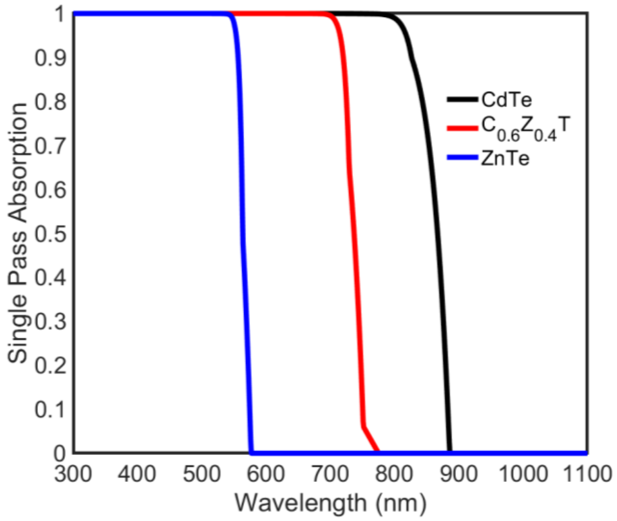

Figure 1. Single-pass absorption spectra for 3 µm thick top cell active absorber (CdTe, $C_{0.6}Z_{0.4}T$ and ZnTe) obeying the Beer-Lambert Law for the incident light as a function of wavelength.

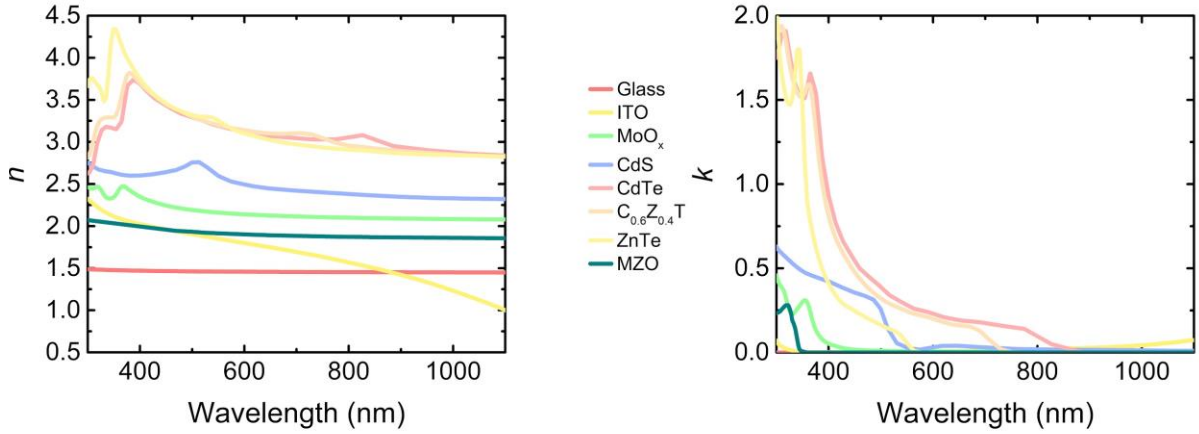

Figure 2. Refractive index (n) and extinction coefficient (k) of materials used in this study.

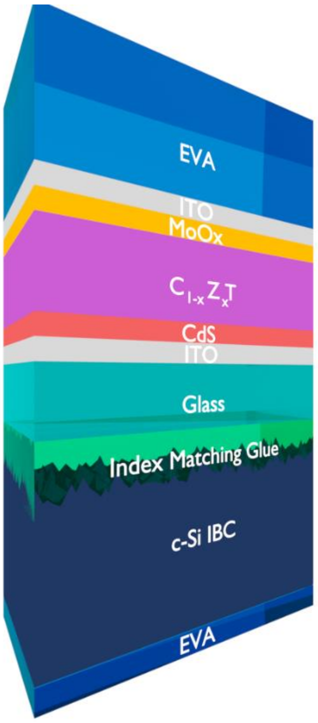

Figure 3. Schematic of the tandem device with inverted top cell structure. Front and rear TCOs are ITO; ETL is CdS; HTL is MoO_x; and an index matching glue is employed between two cells. EVA is used to protect IBC Si cell contacts; glass is employed as the substrate.

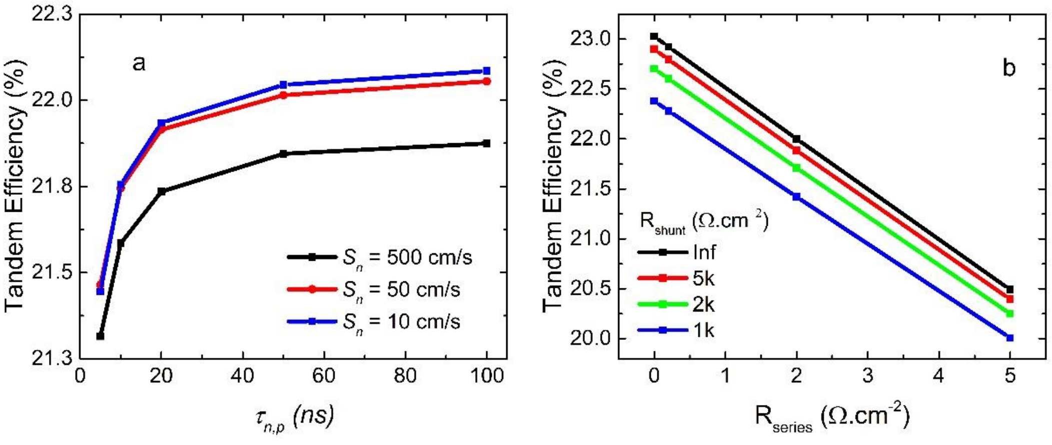

Figure 4. CdTe/IBC Si tandem efficiency as a function of (**a**) lifetime for three surface recombination velocities (10, 50 and 500 cm/s) for a 3 µm CdTe absorber and (**b**) series resistance for four shunt resistances (Infinite, 5k, 2k and 1k Ω/cm^2) for a lifetime of 5 ns and surface recombination velocity of 500 cm/s. CdS is used as the ETL.

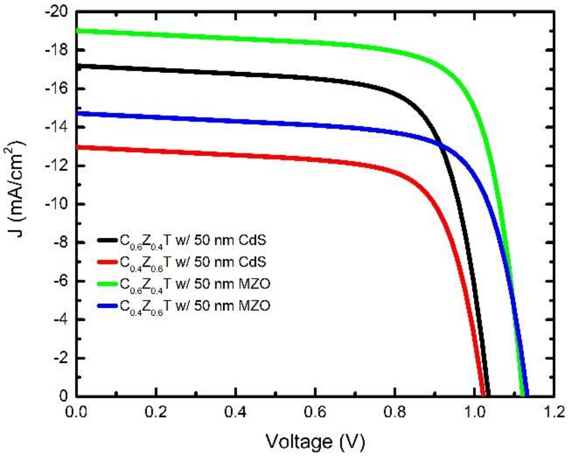

Figure 5. Top cell J-V curves of selected $C_{1-x}Z_xT$ cases (x = 0.4, 0.6) with 50 nm ETL of CdS and MZO.

Table 1. Simulation parameters of solar cells used in SCAPS 1-D.

	CdTe	$C_{0.8}Z_{0.2}T$	$C_{0.6}Z_{0.4}T$	$C_{0.4}Z_{0.6}T$	$C_{0.2}Z_{0.8}T$	ZnTe	CdS	MZO	ITO
E_g (eV)	1.45	1.54	1.69	1.85	2.03	2.19	2.4	3.6	3.72
χ (eV)	4.28	4.23	4.03	3.87	3.69	3.53	4.3	4.2	4.5
CB eff. DOS (cm^{-3})	1.5×10^{18}	1.5×10^{18}	1.5×10^{18}	1.5×10^{18}	1.5×10^{18}	1.5×10^{18}	2.1×10^{18}	2.1×10^{18}	4×10^{19}
VB eff. DOS (cm^{-3})	1.8×10^{19}	1.8×10^{19}	1.8×10^{19}	1.8×10^{19}	1.8×10^{19}	1.8×10^{19}	1.7×10^{19}	1.7×10^{19}	1×10^{18}
V_{te} (cm/V.s)	1×10^7	1×10^7	1×10^7	1×10^7	1×10^7	1×10^7	1×10^7	1×10^7	1×10^7
V_{th} (cm/V.s)	1×10^7	1×10^7	1×10^7	1×10^7	1×10^7	1×10^7	1×10^7	1×10^7	1×10^7
M_e (cm^2/V.s)	700	700	700	700	700	700	70	70	30
M_h (cm^2/V.s)	60	60	60	60	60	60	20	20	5
N_A (cm^{-3})	1×10^{16}	1×10^{16}	1×10^{16}	1×10^{16}	1×10^{16}	1×10^{16}	-	-	-
N_D (cm^{-3})	-	-	-	-	-	-	1.2×10^{18}	1.2×10^{18}	1×10^{21}

E_g: bandgap; χ: electron affinity, DOS: density of states; V_{te}: electron velocity; V_{th}: hole velocity; M_e: electron mobility; M_h: hole mobility; N_A: acceptor density; N_D: donor density.
